# Effect of Coir Fiber Surface Treatment on Interfacial Properties of Reinforced Epoxy Resin Composites

**DOI:** 10.3390/polym14173488

**Published:** 2022-08-25

**Authors:** Shaofeng Ru, Can Zhao, Songmei Yang, Dong Liang

**Affiliations:** Mechanical and Electrical Engineering College, Hainan University, Haikou 570228, China

**Keywords:** coir fiber, interfacial bonding, surface treatment, pullout test, characteristic

## Abstract

Coir-fiber-reinforced epoxy resin composites are an environmentally friendly material, and the use of coir fibers improves the mechanical properties of epoxy resin. In order to improve the interfacial adhesion between coir fibers and the epoxy resin matrix, microwave treatment, alkali treatment, acetic anhydride modification, 3-aminopropyltriethoxysilane modification and their reasonable combination method treatments were carried out on coir fibers, respectively. Scanning electron microscopy (SEM), Fourier transform-infrared (FTIR) and X-ray diffraction (XRD) were used to analyze the effects of the different treatments on the characteristics of the coir fibers, and single-fiber pullout tests were performed on the pullout specimens made from the above coir fibers. The results calculated by the proposed estimation method show that the combination method of alkali treatment and 3-aminopropyltriethoxysilane surface modification could better enhance the interfacial bonding ability between coir fibers and epoxy resin with an interfacial shear strength and pullout energy of 6.728 MPa and 40.237 N·mm, respectively. The principal analysis shows that the method can form both mechanical interlocking and chemical bonds at the interface to enhance the interfacial bonding ability. This study provides a more suitable method for improving the interfacial properties of coir-fiber-reinforced epoxy resin composites and has implications for the study of natural fiber composites.

## 1. Introduction

Nowadays, there is an increased attention toward resources and environmental issues. Natural fibers are being further developed by researchers because of their environmental friendly and renewable characteristics [1,2], and are being used in industries such as textile, construction and automotive industries [3,4,5,6,7]. It is expected that natural fibers will be promoted to more fields in the future [8]. In addition, natural fibers have mechanical properties comparable to synthetic fibers but are less expensive, which is one of the reasons why they are widely used [9,10]. Coir fibers are a common natural fiber, which are derived from waste coconut shells after removing the coconut sap and flesh [11]. Traditionally, most coir fibers were used to make brushes, mats and other daily necessities [12,13]. In recent years, the use of coir fibers as reinforcing fibers to make composite materials has been more extensively researched [14]. Therefore, coir fibers can not only reduce the environmental issues caused by the accumulation and incineration of waste coconut shells, but are also an effective way to reuse coconut waste.

Coir fibers have good mechanical properties, including the best elongation among known natural fibers, as well as the ability to enhance the toughness of epoxy resin. However, coir fibers are hydrophilic, while epoxy resin is hydrophobic. The hydroxyl groups on the surface of coir fibers absorb water molecules to form hydrogen bonds, which prevent the mutual penetration between the two and lead to a poor interfacial bonding ability, which has a negative impact on the mechanical properties of the composites [15,16]. Therefore, the current main research task is to improve the compatibility between coir fibers and the epoxy resin matrix, enhancing the interfacial bonding ability between them [17].

In making natural-fiber-reinforced composites, surface treatments are usually applied to the fibers to improve the interfacial properties between them and the matrix [18]. In addition, the treatments of the fibers can also improve their mechanical properties, such as tensile strength, modulus and elongation [19,20]. Here, the treatment methods are divided into two categories, pretreatment and surface modification, depending on the result of the treatment, where the commonly used pretreatment methods are radiation treatment and alkali treatment, and the commonly used surface modification methods are anhydride surface modification and silane surface modification. Radiation treatment is used to improve the interfacial properties between fibers and the matrix by causing a strong vibration inside the fibers and reducing the polarity of the fiber surfaces. Imoisili et al., investigated the properties of microwave-radiation-treated plantain fiber/MWCNT hybrid epoxy nanocomposites and demonstrated that the microwave-treated fibers could better bond with epoxy resin [21]. Alkali treatment is used to improve the interfacial bonding ability by removing some non-cellulosic substances from the fibers and increasing the surface roughness of the fibers [22]. Shrivastava et al., investigated the effect of alkali treatment on the tensile strength of coir-fiber-reinforced epoxy resin composites. The results show that the coir fibers after alkali treatment were better combined with the matrix, and the tensile strength of the composites was significantly improved compared with previous composites [23]. Anhydride surface modification reduces the number of hydroxyl groups on the fiber surface by replacing them with the group contained in the anhydride to promote the adhesion between the fibers and the matrix, thus improving the interfacial bonding between the coir fibers and the epoxy resin matrix [24,25]. Loong et al., modified flax fibers with acetic anhydride to study its effect on the properties of flax-fiber-reinforced epoxy resin composites. They found that the modified flax fibers improved the interfacial adhesion between the fibers and matrix in the composite [26]. Silane surface modification improves the adhesion between fibers and matrix by chemical bonds, with one end diffusing to connect to the matrix and the other end reacting with the hydroxyl groups on the fiber surface, thus more tightly connecting the two materials [27]. Vijay et al., compared the mechanical properties of leucas aspera fiber epoxy resin composites before and after silane treatment. This research study showed that the ultimate tensile and flexural strength of the composites were improved due to the increased adhesion between the fibers and the matrix after silane treatment [28]. The above single-treatment methods can also be reasonably arranged to form a combined-treatment method. Therefore, it is necessary to choose a method that better enhances the interfacial bonding ability between the fibers and the matrix to make coir-fiber-reinforced epoxy resin composites.

In this study, coir fibers were treated by different methods, and scanning electron microscopy (SEM), Fourier-transform-infrared (FTIR) and X-ray diffraction (XRD) were carried out to analyze the characteristics of the coir fibers after treatment by different methods. Single-fiber pullout tests were performed on the fabricated pullout specimens. The interfacial shear strength and pullout energy were compared to determine a better method for enhancing the interfacial bonding ability between the coir fibers and the matrix, and the reinforcement mechanism of the method was analyzed in terms of the microscopic morphology and chemical elements of the fiber and matrix after pulling. As there are many ways to treat the surface of natural fibers, we treated the coir fibers in different ways and compared various test results to obtain a more suitable method that improved the compatibility between coir fibers and the matrix, so as to better enhance the interfacial bonding properties of coir-fiber-reinforced epoxy resin composites.

## 2. Materials and Methods

### 2.1. Materials

The coir fibers used in the experiment were obtained from coconuts grown in Hainan Province, China. The epoxy resin E-44 used to make the matrix for the pullout specimens was purchased from Guangdong Province, China, with a density of 1150 kg/m^3^, a viscosity of 5000–6000 mPa·s, an epoxy value of 0.41–0.47 eq./100 g and a melting point of 145–155 °C. The chemicals used in this experiment have analytical purity, and included the following: sodium hydroxide (NaOH) (MACKLIN, Shanghai, China), ethanol anhydrous (MACKLIN, Shanghai, China), acetic anhydride (XILONG SCIENTIFIC, Shantou, China), and 3-aminopropyltriethoxysilane (MACKLIN, Shanghai, China).

### 2.2. Preparation of Coir Fibers

#### 2.2.1. Preparation of Coir Fibers Treated by a Single Method

**Figure 1:**
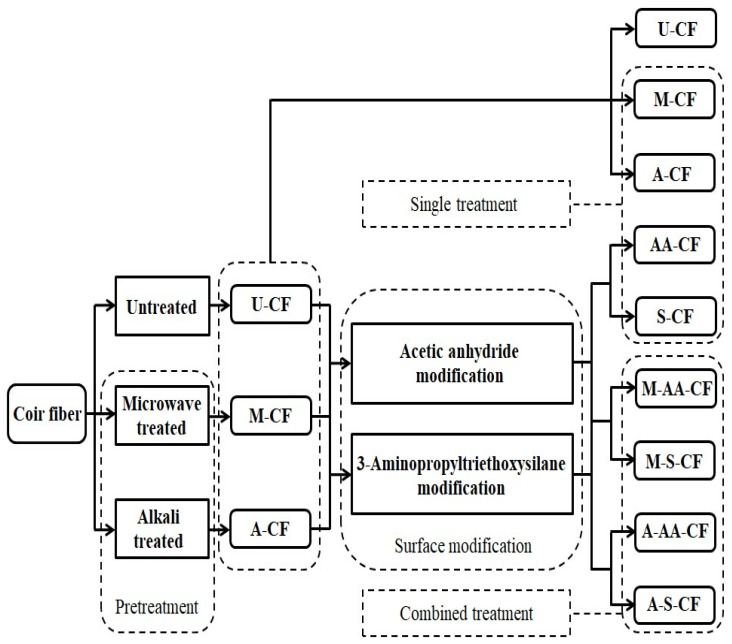
Treatment process of coir fibers.

Coir fibers with similar shapes and diameters were manually selected and soaked in distilled water for 60 min to preliminarily remove the impurities attached to the fiber surface. Then, we washed them repeatedly with distilled water, and cut them into 50 mm after drying. Next, microwave treatment, alkali treatment, acetic anhydride surface modification and 3-aminopropyltriethoxysilane surface modification were applied to coir fibers, respectively. The detailed process is shown in Figure 1, and the treatment conditions of all treatment methods are based on the research of our team and other researchers, as shown in Table 1 [21,22,23,24,25,26,27,28]. After the treatments were completed, these different groups of fibers were soaked in distilled water for 2 h and washed repeatedly. Then, they were placed in a drying oven and dried at 60 °C for 3 h. Thus, four types of coir fibers treated by a single-treatment method were obtained: microwave-treated coir fibers (M-CF), alkali-treated coir fibers (A-CF), acetic-anhydride-modified coir fibers (AA-CF) and 3-aminopropyltriethoxysilane-modified coir fibers (S-CF). Finally, these coir fibers, including untreated coir fibers (U-CF), were separately stored in sealed bags for the subsequent preparation of pullout specimens.

#### 2.2.2. Preparation of Coir Fibers Treated by Combined Method

In the preparation of the coir fibers treated by a combined method, the same process of fiber selection, soaking to remove impurities, repeated washing, drying and then cutting into 50 mm was carried out as described in Section 2.2.1. Next, the coir fibers were treated by combined methods. This detailed process is shown in Figure 1. In the first step, the coir fibers were subjected to microwave treatment and alkali treatment, respectively. After the treatments were completed, they were first soaked in distilled water for 2 h and then dried at 60 °C for 3 h. In the second step, the microwave treated coir fibers and the alkali treated coir fibers were subjected to acetic anhydride surface modification and 3-aminopropyltriethoxysilane surface modification, respectively. After the treatments were completed, they were first soaked in distilled water for 2 h and then dried at 60 °C for 3 h. Thus, four types of coir fibers treated by a combined-treatment method were obtained, which were the coir fibers modified by acetic anhydride after microwave treatment (M-AA-CF), the coir fibers modified by 3-aminopropyltriethoxysilane after microwave treatment (M-S-CF), the coir fibers modified by acetic anhydride after alkali treatment (A-AA-CF), and the coir fibers modified by 3-aminopropyltriethoxysilane after alkali treatment (A-S-CF). Finally, these coir fibers were stored in sealed bags, as were the above-mentioned fibers, in order to prepare pullout specimens for later.

### 2.3. Analysis Method of Coir Fibers Characteristics

#### 2.3.1. Scanning Electron Microscopy

The morphologies of coir fibers treated in different ways were investigated using scanning electron microscopy (SEM) (Verios G4 UC, Thermoscientific, Waltham, MA, USA). The surface morphologies of these 9 groups of coir fibers were observed and analyzed separately at 5 kV to determine their microscopic changes. In addition, the bonded surfaces of the fibers and the epoxy resin matrix were also observed and studied after the coir fibers were pulled out. Prior to these, the coir fibers and epoxy resin matrix needed to be gold-sprayed to make them conductive and ensure the smooth observation under SEM.

#### 2.3.2. Fourier Transform-infrared Spectroscopy

The coir fibers treated in different ways were investigated and analyzed by Fourier-transform infrared spectrometer (FTIR) (T27, Bruker, Billerica, Germany). After drying the coir fiber powder, 2 mg of coir fiber powder and 300 mg of potassium bromide (KBr) were thoroughly ground and mixed under infrared lamp baking, and then poured into a mold and extruded to make a disc-shaped specimen for testing. All of the above nine types of coir fiber needed to go through this process to make FTIR test specimens. The data displayed by the spectra were recorded in the wavenumbers range of 400 to 4000 cm^−1^ with a resolution of 4 cm^−1^. Each disk-shaped test sample was placed into the instrument, and the infrared spectra of samples can be obtained after scanning. Finally, the infrared spectra were baseline corrected, scaled and smoothed for subsequent analysis.

#### 2.3.3. X-ray Diffraction

The crystallinity index (CrI) of coir fibers treated in different ways was measured by X-ray diffractometer (XRD) (Smart Lab, Rigaku, Tokyo, Japan). The coir fibers of each group were successively crushed into fine particles by a pulverizer to prepare the test samples. The samples of each group were sequentially scanned in the scanning range 2θ of 5–60° at a scanning speed of 5°/min. After the XRD spectra were obtained, the relative CrI of each group of coir fibers was calculated using the Segal empirical method based on the data of the spectra [29].

(1) $$\mathrm{CrI}=\frac{I_{002}-I_{\mathrm{am}}}{I_{002}}\;\times \;100\%$$

where CrI is crystallinity index, I_002_ is the maximum intensity of 002 lattice diffraction peak at a 2θ close to 22° and I_am_ is the minimum intensity diffraction of amorphous materials at a 2θ close to 18°.

### 2.4. Preparation of Pullout Specimens

**Figure 2:**
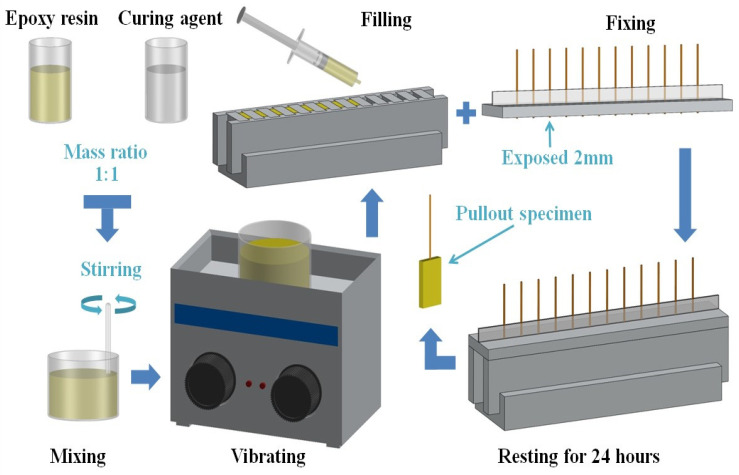
Production process of pullout specimens.

According to the process shown in Figure 2, the pullout specimens were prepared with the above 9 groups of coir fibers. The epoxy resin was mixed 1:1 with the curing agent, stirred well, and then placed in an ultrasonic vibrator for 5 min, which made the internal bubbles move up to the surface to be gradually removed. After that, the epoxy resin was extracted with a syringe and injected into the special mold. Meanwhile, the coir fibers were fixed on the mold cover, exposing the lower end of the fibers by 2 mm to ensure that each coir fiber entered into the epoxy resin matrix by 2 mm. Then, the cover was reunited with the mold and left to stand for 24 h. When the epoxy resin was solidified and formed, the mold was removed to obtain the final pullout specimens. Finally, according to the coir fibers used, the completed pullout specimens were named U-CFPS, M-CFPS, A-CFPS, AA-CFPS, S-CFPS, M-AA-CFPS, M-S-CFPS, A-AA-CFPS and A-S-CFPS, respectively.

### 2.5. Single-Fiber Pullout Test

The single-fiber pullout tests were conducted on the pullout specimens using an Electronic Universal Testing Machine (3343, INSTRON, Boston, MA, USA) to compare and analyze the interfacial bonding ability between nine different groups of coir fibers and epoxy resin. Specifically, the epoxy resin block below the pullout specimen was clamped with the lower collet of the instrument, and the coir fiber located at the upper end needed to be fixed to the upper collet of the instrument by a special fixture, while keeping the fiber in a vertical position. After the pullout specimen was clamped, it was stretched at a speed of 2 mm/min. The pullout load–displacement curves obtained from the tests were used to calculate the interfacial shear strength and estimate the pullout energy. Each group of tests was repeated 10 times, and the average values were taken to characterize the interfacial bonding ability of pullout specimens.

### 2.6. Processing Methods of Test Data

**Figure 3:**
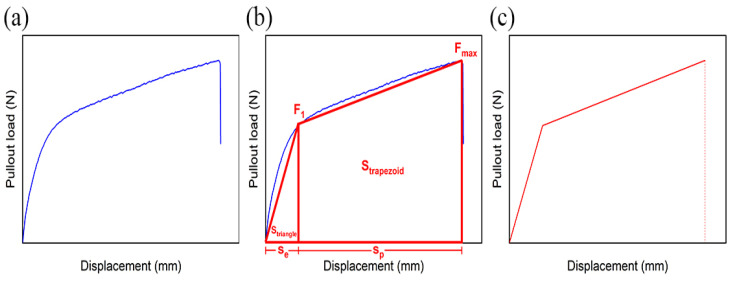
(**a**) Approximate shape of load–displacement curve obtained in the test, (**b**) estimation method of load–displacement curve, and (**c**) approximate shape of load–displacement curve obtained after estimation.

In order to investigate the effect of different treatment methods on the interfacial bonding ability between coir fibers and epoxy resin matrix, the interfacial shear strength and pullout energy obtained from the single-fiber pullout test were used to characterize it. The interfacial shear strength can be calculated from the maximum pullout load in the test. Because the diameter of each coir fiber varies, the size of its diameter will be especially different after different methods of treatment. In order to more accurately calculate the interfacial shear strength, a Stereomicroscope (M205 FA, LEICA, Wetzlar, Germany) was used to take photos and make measurements. Each fiber was measured once in each of two mutually perpendicular directions, and the average value was taken as the diameter. In addition, the pullout energy can be estimated from the area enclosed by the load–displacement curve. The approximate shape of the load–displacement curve obtained from the single-fiber pullout test of coir fibers is shown in Figure 3a. To facilitate the calculation and comparative analysis, an estimation method is proposed here as shown in Figure 3b [30]. Specifically, since this load–displacement curve is divided into an elastic deformation phase and a plastic deformation phase, they are estimated to have a right triangle and a right trapezoid. In this way, the area enclosed by the load–displacement curve can be obtained from the sum of the areas of the right triangle and the right trapezoid, as shown in Equation (2). The shape of the load–displacement curve estimated by this method is shown in Figure 3c.

(2) $${S=S}_{\mathrm{triangle}}{+S}_{\mathrm{trapezoid}}$$

where S is the area enclosed by the load–displacement curve, S_triangle_ is the area of the right triangle and S_trapezoid_ is the area of the right trapezoid.

## 3. Results and Discussion

### 3.1. Scanning Electron Microscopy Observation and Analysis

**Figure 4:**
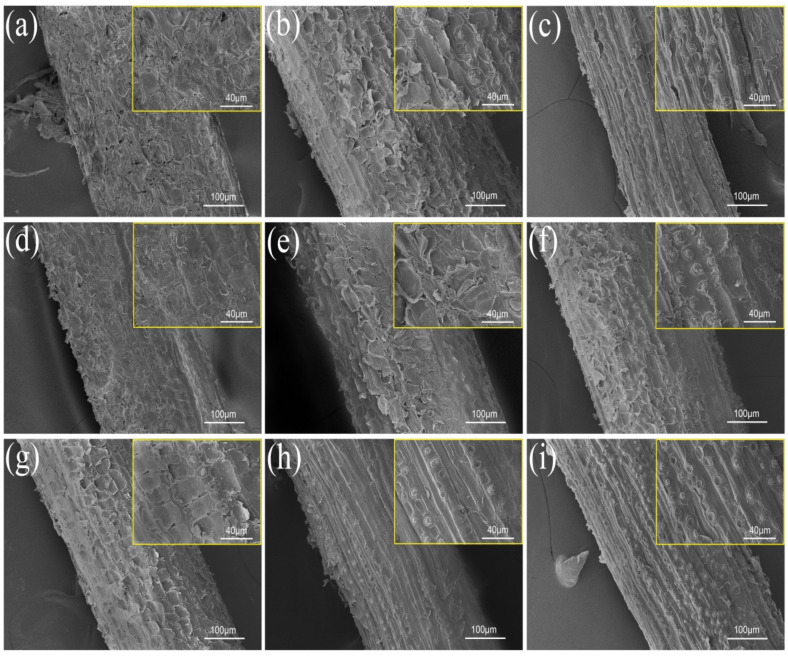
SEM micrographs of coir fibers: (**a**) U-CF, (**b**) M-CF, (**c**) A-CF, (**d**) AA-CF, (**e**) S-CF, (**f**) M-AA-CF, (**g**) M-S-CF, (**h**) A-AA-CF, (**i**) A-S-CF.

Figure 4 shows SEM micrographs of the surfaces of untreated and differently treated coir fibers, and in order to observe some detailed features more clearly, SEM micrographs of coir fibers with a higher magnification are also shown in the figure. The untreated coir fiber is shown in Figure 4a, and the surface of U-CF is wrapped with a large amount of non-cellulose substances, such as lignin, pectin and impurities. Due to their presence, the surface of coir fiber looks rougher, but in fact, these substances are only attached to the surface of coir fiber. It can be observed from the magnified picture that their structure is loose and interposed between the fiber and the matrix, which weakens the interfacial bonding ability, and thus debonding can easily occur [23,31]. These substances are reduced in M-CF, as shown in Figure 4b. Some areas on the surface of M-CF show a lamellar shape, which is due to the internal vibration of the fiber caused by microwave radiation of the coir fiber and the resulting damage to the fiber surface organization. This enhances the surface roughness of coir fibers and plays a certain role in improving the interfacial bonding ability between the fibers and the matrix [21]. As shown in Figure 4c, compared with U-CF and M-CF, the surface of A-CF looks more shriveled, because the coir fiber removes most of the non-cellulosic substances attached to its surface after soaking in NaOH solution, and forms some bulges and grooves [32,33]. This structure can be clearly seen in the SEM micrograph with a high magnification of the A-CF surface. When the epoxy resin matrix material penetrates into this bumpy surface structure, it helps to form a mechanical interlock between the fibers and the matrix, so as to improve the interfacial bonding ability between them [34].

The next SEM micrographs are obtained after different surface modifications of U-CF, M-CF and A-CF, where Figure 4d–i show the observation effect of AA-CF, S-CF, M-AA-CF, M-S-CF, A-AA-CF and A-S-CF, respectively. From the figures, it can be seen that the non-cellulose substances attached to the surface of coir fibers were reduced to different degrees after treatment by different methods, among which, A-S-CF showed the clearest reduction. The presence of impurities on its surface can hardly be seen from the magnified picture. As a whole, its surface showed irregular grooves and a large number of unevenly distributed bulges and pits. For A-S-CF, this uneven surface structure has advantages in improving the interfacial bonding ability between fiber and matrix [35]. On the one hand, it helps to form mechanical interlocking, which can significantly improve the interfacial compatibility between coir fiber and epoxy resin matrix. On the other hand, it facilitates the hook-up of 3-aminopropyltriethoxysilane, which acts as an intermediary to connect the coir fiber to the epoxy resin matrix by chemical bonding and thus improves interfacial interactions.

### 3.2. Fourier Transform-Infrared Spectroscopy Analysis

**Figure 5:**
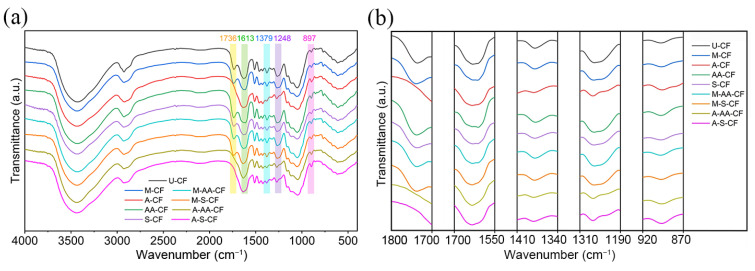
(**a**) FTIR spectra of coir fibers, (**b**) Enlarged view of labeled regions in FTIR spectra.

The FTIR spectra of U-CF, M-CF, A-CF, AA-CF, S-CF, M-AA-CF, M-S-CF, A-AA-CF and A-S-CF are presented in Figure 5a, and the labeled regions in the spectra are enlarged for easy observation and analysis, as shown in Figure 5b. The change in the absorption peak located at 1736 cm^−1^ is related to the C=O stretching vibration in the acetyl group of hemicellulose. The disappearance of the absorption peaks of A-CF, A-AA-CF and A-S-CF at this location indicates that most of the hemicellulose was removed from the coir fibers after alkali treatment [36]. While the intensity of AA-CF and M-AA-CF increases slightly at this peak, which is caused by the emergence of anhydride groups after the surface modification of coir fibers with acetic anhydride [24]. The absorption peak at 1613 cm^−1^ originates from the C=C stretching vibration in aromatic lignin, and the decrease in the intensity at this location can be attributed to the removal of lignin from the coir fibers [8]. The absorption peak at 1379 cm^−1^ is caused by a C-H bending vibration, and the changes in the peak value are related to lignin. The absorption peak at 1248 cm^−1^ is related to the C-O-C stretching vibration in lignin. The intensities of A-CF, A-AA-CF and A-S-CF significantly decrease at this peak, which explains how most lignin is removed from coir fibers after alkali treatment [32,37]. The absorption peak at 897 cm^−1^ is related to the C-H rocking vibration in cellulose, and the increase in the intensity of the absorption peak at this location corresponds to the increased level of cellulose after the removal of non-cellulosic substances [38].

In addition, no absorption peaks specific to the 3-aminopropyltriethoxysilane treated coir fibers appeared on the spectra. When high concentrations of silane solution are used, the corresponding absorption peaks appear, but their intensity is also weak. Too high a concentration of silane solution will not completely react and will form aggregates between the coir fibers and the epoxy resin matrix, affecting the interfacial bonding effect between them [27]. The concentration of the silane solution chosen in the experiment is only 5%; therefore, the intensity of the absorption peak may be too weak and not easily visible.

### 3.3. X-ray Diffraction Analysis

**Figure 6:**
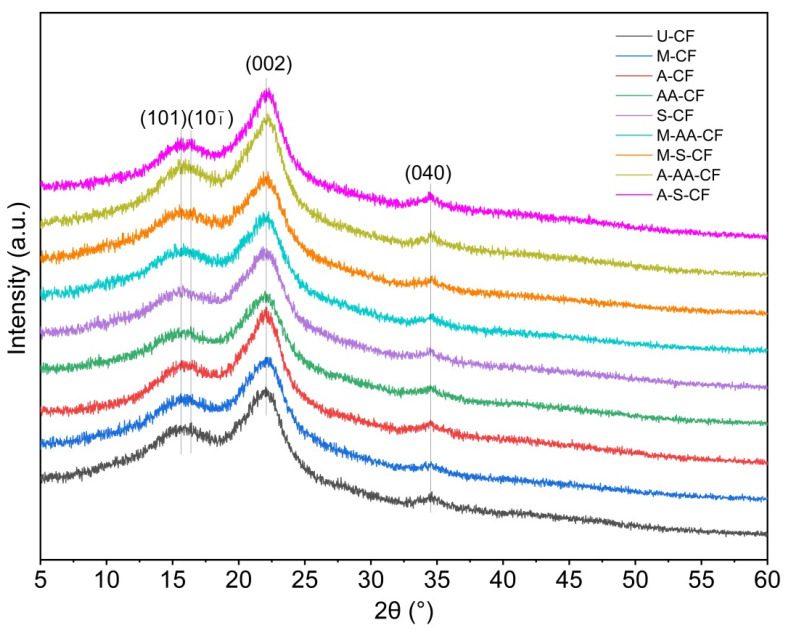
XRD spectra of coir fibers.

The X-ray diffraction patterns of U-CF, M-CF, A-CF, AA-CF, S-CF, M-AA-CF, M-S-CF, A-AA-CF and A-S-CF are shown in Figure 6. From this figure, it can be seen that the coir fibers treated by different methods have XRD spectra with similar shapes and all show distinct peaks around the 2θ angles of 16°, 22° and 35°. This indicates that these methods did not change the cellulose I crystal structure of the coir fibers [39]. The CrI values of the coir fiber samples calculated by the Segal empirical method are shown in Table 2. Through the comparative data, it was found that the CrI value of U-CF is only 34.6%, while the CrI values of the other treated coir fibers increased to varying degrees. Among them, A-CF, A-AA-CF and A-S-CF significantly increased, especially the CrI value of A-S-CF, which reached 42.3% [40]. This is mainly because most of the hemicellulose, lignin and pectin on the fiber surface was removed, increasing the proportion of cellulose [41]. The increase in CrI value helps to improve the mechanical properties of the coir fibers themselves, and at the same time, the removal of non-cellulose substances has a positive effect on improving the interfacial bonding ability between the coir fibers and the epoxy resin matrix. This is consistent with the observation results of SEM and the analysis results of FTIR.

### 3.4. Single-Fiber Pullout Test Results

During the single-fiber pullout test, the coir fiber pullout specimen was fixed on the collet of the tensile testing machine, and the pullout load was applied on the end of the coir fiber in the pullout specimen. As the test proceeded, the coir fiber was pulled out from the epoxy matrix when the stress exceeded the interfacial shear strength of the pullout specimen.

**Figure 7:**
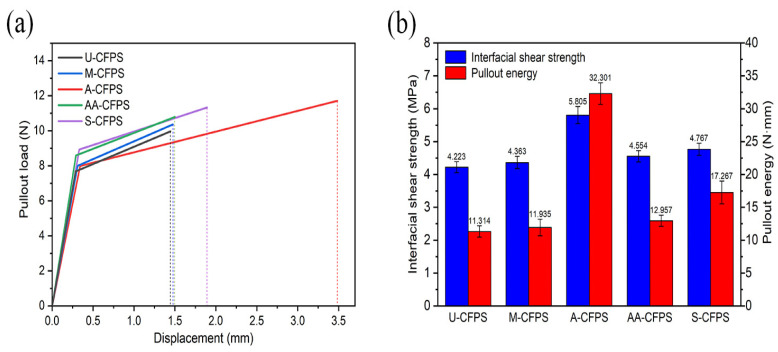
Single-fiber pullout test results for untreated and single method treated coir fiber pullout specimens: (**a**) Load–displacement curves, (**b**) The calculated interfacial shear strength and pullout energy.

In order to investigate the effect of different single treatment methods on the interfacial bonding between coir fibers and the epoxy resin matrix, two kinds of pullout specimens (M-CFPS and A-CFPS) with only pretreated coir fibers and two kinds of pullout specimens (AA-CFPS and S-CFPS) with only surface-modified coir fibers were selected for a single-fiber pullout test, and the single-fiber pullout test of U-CFPS was used as a comparison. The resulting load–displacement curves for each group are estimated by the method described in Section 2.6, as shown in Figure 7a. As can be seen from the figure, the maximum pullout load for U-CFPS is less than 10N, while for M-CFPS, A-CFPS, AA-CFPS and S-CFPS, larger pullout loads are required to separate the coir fibers from the epoxy resin matrix. A-CFPS has the largest pullout load in the pullout specimens made of coir fibers treated by single-treatment methods. Because the coir fibers undergo different changes after different treatments, they are further analyzed by calculating the interfacial shear strength and pullout energy using Equations (3) and (4), respectively [17,30,42]. The key data used for the calculations are shown in Table 3.

(3) $$\tau =\frac{F_{\max}}{d\pi l}$$

where τ is interfacial shear strength, F_max_ is the maximum load during the pullout process, d is the average diameter of coir fibers, and l is the depth of coir fibers buried in the epoxy resin matrix.

(4) $$E=\frac{F_{1}{\times (s}_{e}{+s}_{p}{)+F}_{\max}{\times s}_{p}}{2}$$

where E is pullout energy, F_1_ the maximum load in the elastic deformation phase of the pullout process, s_e_ is the displacement of the elastic deformation phase during the pullout process, F_max_ is the maximum load during pullout process and s_p_ is the displacement of the plastic deformation phase during the pullout process.

Figure 7b shows the calculated results. It is apparent that U-CFPS exhibits a weak interfacial bonding ability, its interfacial shear strength is only 4.223 MPa, and its pullout energy is only 11.314 N·mm. Compared with U-CFPS, the interfacial shear strength and pullout energy of M-CFPS, AA-CFPS and S-CFPS slightly increase, while A-CFPS shows a significant increase, with an interfacial shear strength of 5.805 MPa and a pullout energy of 32.301 N·mm, which are 37.462% and 185.496% higher than U-CFPS, respectively. This is principally caused by the alkali treatment because the soaking of coir fibers in NaOH solution removes the impurities attached to their surfaces and gives the fibers more direct contact with the matrix. At the same time, it also partially removes the hemicellulose, lignin, pectin and other substances contained in the fibers, improves the compatibility of the two materials, and enhances the interfacial bonding between the fibers and the matrix material [43]. In this way, not only does the maximum pullout load increase, but the diameter also decreases after alkali treatment; therefore the interfacial shear strength of the pullout specimen is improved. In addition, elongation is an advantageous property of coir fibers when compared with other natural fibers, and the alkali treatment makes this advantageous property more prominent. Therefore, more pullout energy is required to pull the fibers out of the epoxy resin matrix.

**Figure 8:**
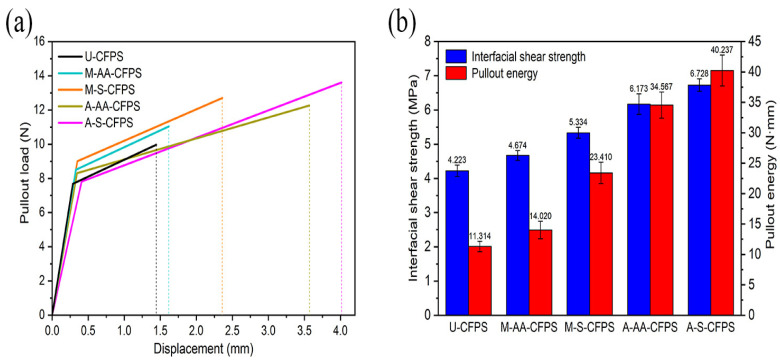
Single-fiber pullout test results for untreated and combined method treated coir fiber pullout specimens: (**a**) Load–displacement curves, (**b**) The calculated interfacial shear strength and pullout energy.

Among these single-treatment methods for coir fibers, alkali treatment showed a prominent effect on improving the interfacial bonding ability between coir fibers and epoxy resin matrix. In order to further improve the interfacial bonding ability, the combined-treatment methods of coir fibers were explored. Four pullout specimens made of coir fibers treated with a combination of pretreatment and surface modification (M-AA-CFPS, M-S-CFPS, A-AA-CFPS and A-S-CFPS) were selected for single-fiber pullout tests, and U-CFPS was used as a control test. After the tests were completed, a better combination of treatment methods was compared and analyzed according to the data obtained after the coir fibers were pulled out of the epoxy resin matrix [42]. Figure 8a shows the load–displacement curves generated by each group estimated by the same method as above. From the figure, it can be seen that the maximum pullout load of the pullout specimens made from the combined treated coir fibers has increased to different degrees compared to the pullout specimens made from the untreated coir fibers, and the maximum pullout load of A-S-CFPS is the largest. According to the data in Table 4, the interfacial shear strength and pullout energy calculated by Equations (3) and (4) are shown in Figure 8b, which more intuitively show the interfacial bonding ability of the specimens. It can be seen that the pullout specimens made of coir fibers with the combination treatment have a better interfacial bonding ability than the previous specimens, and the interfacial shear strength and pullout energy of A-S-CFPS are the largest, with values of 6.728 MPa and 40.237 N·mm, respectively. In addition, two aspects can be analyzed. On the one hand, they were divided into two groups. The interfacial shear strength and pullout energy of the two specimens in the first group (M-AA-CFPS and M-S-CFPS) and the second group (A-AA-CFPS and A-S-CFPS) were compared, respectively. It can be seen that the interfacial bonding ability of the pullout specimen made from coir fibers modified by 3-aminopropyltriethoxysilane is better than that of the pullout specimen made from coir fibers modified by acetic anhydride. It may be that the surface modification of 3-aminopropyltriethoxysilane not only consumes the hydroxyl group but also forms a more stable carboxyl group to connect with the matrix, demonstrating a better interfacial bonding ability. On the other hand, they were also divided into two groups. The interfacial shear strength and pullout energy of the two specimens in the first group (M-AA-CFPS and A-AA-CFPS) and the second group (M-S-CFPS and A-S-CFPS) were compared, respectively. It can be seen that the pullout specimens made from coir fibers using alkali treatment as a pretreatment have better interfacial bonding ability. Firstly, this is because alkali treatment removes most of the non-cellulosic substances on the surface of coir fibers, which helps to form mechanical interlocking while reducing the occurrence of debonding. Furthermore, the presence of impurities on the surface of coir fibers does not facilitate the reaction between fibers and acetic anhydride or 3-aminopropyltriethoxysilane, while the presence of bulges and grooves on the surface of fibers facilitates the attachment of these substances.

### 3.5. Interfacial Characteristic Analysis

The purpose of treating coir fibers in different ways is to compare and obtain a method that can make coir fibers bond more closely with the epoxy resin matrix. From the results of the above single-fiber pullout test, it can be concluded that all the methods in the test can enhance the interfacial bonding between the coir fibers and the epoxy resin matrix. This implies that the results of this experiment are consistent with those of other studies in the literature, and they enhance the interfacial properties of the composites, which are of interest for the study of composites [17]. In the test, the combination of alkali treatment and 3-aminopropyltriethoxysilane surface modification was the most effective treatment method compared to the other methods. Our calculation shows that the interfacial shear strength and pullout energy of A-S-CFPS are 59.318% and 255.639% higher than those of U-CFPS, respectively. In order to further study the influence mechanism of this method on interfacial bonding, the interfacial characteristics of A-S-CFPS and U-CFPS were compared and analyzed.

**Figure 9:**
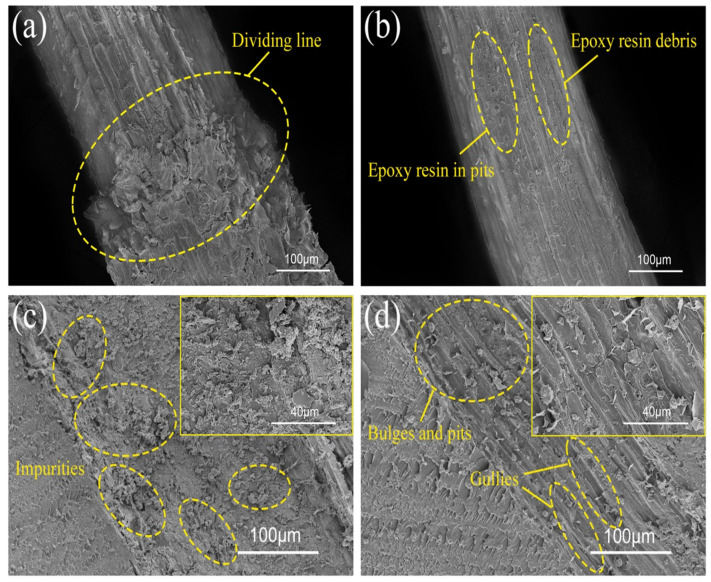
SEM micrographs of interfaces after completing the single-fiber pullout test: (**a**) U-CF, (**b**) A-S-CF, (**c**) matrix of U-CFPS, and (**d**) matrix of A-S-CFPS.

Figure 9 shows the SEM micrographs of coir fibers and the epoxy resin matrix after completing the single-fiber pullout test. The coir fiber in Figure 9a is from U-CFPS, and the existence of a dividing line can clearly be seen, where the thicker end was originally exposed to the exterior of the matrix, while the thinner end was originally bonded to the matrix. The figure shows a thick layer of pectin and impurities attached to the surface of U-CF, which are not tightly bound to each other, and the separation of these substances during fiber pulling causes debonding to occur [23,31]. For the coir fiber pulled out from A-S-CFPS (Figure 9b), no clear dividing line was found, and the bonded epoxy resin matrix debris could be seen near the bulges and pits on the fiber surface. Figure 9c is the SEM micrograph of the interface on the epoxy resin matrix after the U-CF is pulled out, and the interface is relatively flat from the overall view. After magnification, it can be seen that the interface is covered with some substances, which come from the surface of U-CF. On the contrary, there are a lot of gullies on the interface bonded with A-S-CF (Figure 9d), and bulges and pits with similar characteristics to the surface of A-S-CF can be observed in the enlarged micrograph, which is formed after the fluid epoxy resin enters the bulges and pits on the fiber surface and solidifies. Figure 9 fully illustrates that the substances attached to the surface of U-CF are not conducive to the bonding between coir fibers and the matrix, while the removal of these substances creates a mechanical interlock between A-S-CF and the matrix, which makes the bonding between them tighter [35].

**Figure 10:**
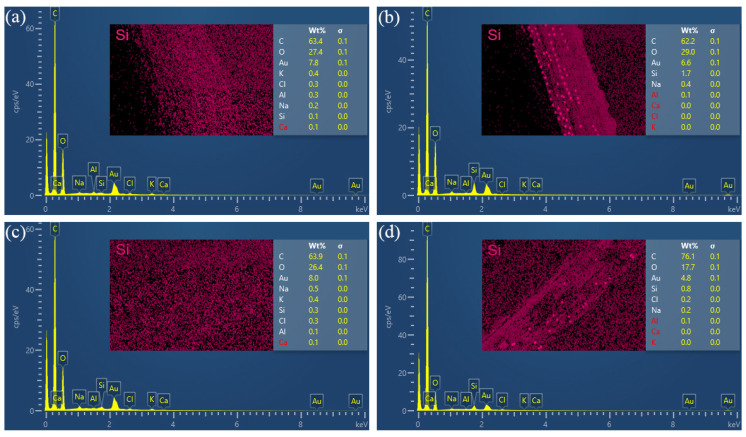
The percentage content of each element and the distribution of Si element: (**a**) U-CF, (**b**) A-S-CF, (**c**) Matrix of U-CFPS, (**d**) Matrix of A-S-CFPS.

**Figure 11:**
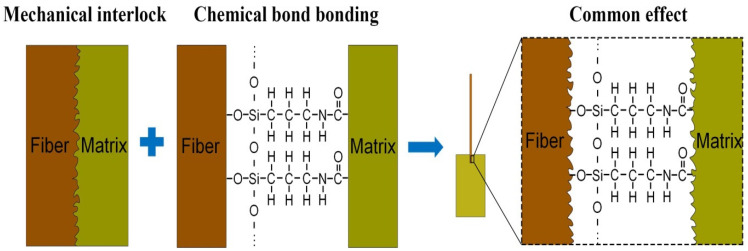
Enhancement mechanism of interfacial properties by the combination of alkali treatment and 3-aminopropyltriethoxysilane modification.

Both coir fibers and the epoxy resin matrix contain only C, H, and O elements, and impurities attached to them contain small amounts of other elements, while 3-aminopropyltriethoxysilane contains Si elements; therefore, it is analyzed by studying the information about chemical elements on the interface. The percentage content of each element and the distribution of Si element provided in Figure 10a–d are from the fiber surface in U-CFPS, the fiber surface in A-S-CFPS, the matrix interface in U-CFPS and the matrix interface in A-S-CFPS, respectively. Through comparison, it was found that in U-CFPS, the proportion of Si element content on the fiber surface and the interface of the matrix are 0.1% and 0.3%, respectively, while in A-S-CFPS, the proportion of Si element content on the fiber surface and the interface of the matrix are 1.7% and 0.8%, respectively. In addition, there is no obvious aggregation of Si elements on the Si element distribution diagram of the fiber surface and matrix interface in U-CFPS, while the contour of coir fiber can be clearly seen on the Si element distribution diagram of the fiber surface and matrix interface in A-S-CFPS. There are bright spots produced by a large amount of aggregation of Si elements in both displayed contours. This shows that the surface modification of coir fibers by 3-aminopropyltriethoxysilane not only increases the Si element content on the fiber surface but also increases the Si element content at the matrix interface in contact with it. This fully illustrates that the 3-aminopropyltriethoxysilane tightens the bond between the coir fibers and the epoxy resin matrix by producing chemical bonds [44,45]. Therefore, A-S-CFPS has a better interfacial bonding ability, which is the result of the combined effect of mechanical interlocking and chemical bonding between the coir fiber and the epoxy resin matrix (Figure 11).

## 4. Conclusions

In this study, the effects of four single treatment methods and four combined-treatment methods of coir fibers on the interfacial bonding ability between coir fibers and epoxy resin matrix were compared based on the characteristic analysis of coir fibers and single-fiber pullout tests, with the aim of devising a better method. The results of single-fiber pullout tests on different pullout specimens show that all of these methods can enhance the interfacial bonding between the coir fibers and the epoxy resin matrix. Through comparison, it was concluded that A-S-CFPS had a better interfacial bonding ability. Its interfacial shear strength and pullout energy are 6.728 MPa and 40.237 N·mm, respectively, which are 59.318% and 255.639% higher compared to U-CFPS. By observing the microscopic morphology of the fibers and matrix after pullout, it was found that the coir fiber in A-S-CFPS removed the non-cellulose substances and formed mechanical interlocking with the matrix, thus improving the interfacial properties. By analyzing the chemical elements on the fibers and matrix after pullout, it was found that the chemical bonds generated by the 3-aminopropyltriethoxysilane in A-S-CFPS made the fiber bond more tightly with the matrix. Therefore, the combination of alkali treatment and 3-aminopropyltriethoxysilane surface modification can better improve the interfacial bonding ability between coir fibers and the epoxy resin matrix.

## Figures and Tables

**Table 1:** Conditions of each treatment method.

Categories	Treatment Methods	Treatment Conditions		
Pretreatment	Microwave treatment	Power (W)	Frequency (MHz)	Time (min)
		700	2450	5
	Alkali treatment	Concentration (%)	Time (hours)	Temperature (°C)
		5	15	30
Surface modification	Acetic anhydride modification	Concentration (%)	Time (min)	Temperature (°C)
		5	60	30
	3-Aminopropyltriethoxysilane modification	Concentration (%)	Time (min)	Temperature (°C)
		5	60	30

**Table 2:** Crystallinity index values of coir fibers.

Crystallinity Index (%)								
U-CF	M-CF	A-CF	AA-CF	S-CF	M-AA-CF	M-S-CF	A-AA-CF	A-S-CF
34.6 (±0.09)	35.0 (±0.04)	41.2 (±0.18)	35.4 (±0.11)	36.6 (±0.09)	35.9 (±0.11)	36.7 (±0.14)	41.4 (±0.15)	42.3 (±0.18)

**Table 3:** Key data for calculating interfacial shear strength and pullout energy of untreated and single method treated coir fiber pullout specimens.

Notation	F_1_ (N)	s_e_ (mm)	F_max_ (N)	s_p_ (mm)	d (mm)
U-CFPS	7.685 (±0.410)	0.287 (±0.013)	9.967 (±0.364)	1.157 (±0.084)	0.376 (±0.010)
M-CFPS	7.999 (±0.405)	0.312 (±0.015)	10.363 (±0.444)	1.164 (±0.122)	0.378 (±0.007)
A-CFPS	7.995 (±0.372)	0.342 (±0.015)	11.708 (±0.534)	3.140 (±0.163)	0.321 (±0.006)
AA-CFPS	8.595 (±0.490)	0.291 (±0.015)	10.787 (±0.408)	1.208 (±0.100)	0.377 (±0.006)
S-CFPS	8.928 (±0.423)	0.332 (±0.021)	11.322 (±0.445)	1.559 (±0.177)	0.378 (±0.005)

**Table 4:** Key data for calculating interfacial shear strength and pullout energy of untreated and combined method treated coir fiber pullout specimens.

Notation	F_1_ (N)	s_e_ (mm)	F_max_ (N)	s_p_ (mm)	d (mm)
U-CFPS	7.685 (±0.410)	0.287 (±0.013)	9.967 (±0.364)	1.157 (±0.084)	0.376 (±0.010)
M-AA-CFPS	8.508 (±0.348)	0.329 (±0.011)	11.043 (±0.320)	1.291 (±0.135)	0.376 (±0.004)
M-S-CFPS	9.007 (±0.538)	0.349 (±0.018)	12.701 (±0.395)	2.012 (±0.169)	0.379 (±0.006)
A-AA-CFPS	8.311 (±0.423)	0.345 (±0.015)	12.256 (±0.457)	3.222 (±0.157)	0.316 (±0.007)
A-S-CFPS	7.820 (±0.411)	0.408 (±0.018)	13.612 (±0.342)	3.606 (±0.184)	0.322 (±0.004)

## Data Availability

Not applicable.
